# Biodegradation of Polyethoxylated Nonylphenols

**DOI:** 10.1155/2013/284950

**Published:** 2013-07-10

**Authors:** Yassellis Ruiz, Luis Medina, Margarita Borusiak, Nairalith Ramos, Gilberto Pinto, Oscar Valbuena

**Affiliations:** ^1^Centro de Investigaciones Microbiológicas Aplicadas (CIMA), Facultad de Ciencias de la Salud, Universidad de Carabobo, Valencia 2005, Venezuela; ^2^Centro de Investigaciones Químicas (CIQ), Facultad de Ingeniería, Universidad de Carabobo, Valencia 2005, Venezuela; ^3^Departamento de Química, Facultad de Ciencias y Tecnología (FACYT), Universidad de Carabobo, Bárbula 2001, Naguanagua 2005, Carabobo, Venezuela

## Abstract

Polyethoxylated nonylphenols, with different ethoxylation degrees (NPEO_*x*_), are incorporated into many commercial and industrial products such as detergents, domestic disinfectants, emulsifiers, cosmetics, and pesticides. However, the toxic effects exerted by their degradation products, which are persistent in natural environments, have been demonstrated in several animal and invertebrate aquatic species. Therefore, it seems appropriate to look for indigenous bacteria capable of degrading native NPEO_*x*_ and its derivatives. In this paper, the isolation of five bacterial strains, capable of using NPEO_*15*_, as unique carbon source, is described. The most efficient NPEO_*15*_ degrader bacterial strains were identified as *Pseudomonas fluorescens* (strain Yas2) and *Klebsiella pneumoniae* (strain Yas1). Maximal growth rates were reached at pH 8, 27°C in a 5% NPEO_*15*_ medium. The NPEO_*15*_ degradation extension, followed by viscometry assays, reached 65% after 54.5 h and 134 h incubation times, while the COD values decreased by 95% and 85% after 24 h for the Yas1 and Yas2 systems, respectively. The BOD was reduced by 99% and 99.9% levels in 24 h and 48 h incubations. The viscosity data indicated that the NPEO_*15*_ biodegradation by Yas2 follows first-order kinetics. Kinetic rate constant (*k*) and half life time (*τ*) for this biotransformation were estimated to be 0.0072 h^−1^ and 96.3 h, respectively.

## 1. Introduction

Alkyl polyethoxylates (APEO), widely used as industrial and domestic surfactants, are added to a variety of products such as dispersants, emulsifiers, detergents, dyes, antioxidants, pesticides, spermicides, and cosmetics [1–4]. Most of these compounds including nonylphenol polyethoxylates (NPEO_*x*_) are incorporated to aqueous solutions, and after being used, they are discharged in industrial or municipal water waste and eventually enter water treatment plants [5, 6]. Due to their persistency at low temperatures NPEO_*x*_ remain in the environment and could be bioaccumulated, which is harmful to animals, humans, and other biological aquatic species [7–10], as its degradation products are more toxic than the original molecule [1, 3, 8, 11–13]. The toxic effects exerted by NPEO_*x*_ and its degradation products include reduction of spermatozoid number, increase of testicular cancer, and feminism in aquatic male species [7, 8, 14, 15]. The highly ethoxylated NPEO_*x*_ compounds lack estrogenic activity, whereas the low ethoxylated ones, included nonylphenol, which arise from nonylphenol ethoxylates by degradation in natural environments, do affect fishes, amphibians, birds, mammalians, invertebrates species such as crustaceans, mollusks, algae, yeast, and plants [3, 16–19]. These compounds also negatively affect microbial biomass growth by competitive inhibition mechanisms when the biomass is not acclimated [20]. Apparently, the estrogenic activity is induced because nonylphenol mimics the structure of the female sexual hormones, the estrogens [5, 21, 22]. Thus, nonylphenol and ethoxylated alkylphenols have been classified as pseudoestrogens and endocrine disrupters because of the harmful effects on the endocrine systems, the reproductive cycles, and other vital functions in humans and other animal species [4, 6, 13, 23, 24].

NPEO_*x*_ degradation occurs through several pathways: (I) Cleavage of the ether bonds with the generation of alkyl-phenol derivatives with shorter ethoxylated chains, which yields mono- and diethylated compounds (NPEO_1_ and NPEO_2_) that could further be carboxylated to form ethoxyacetic and acetic nonylphenol acids [11, 25–27]. (II) *ω*-Carboxylation of the ethoxylated chain, yielding different polyethoxylated derivatives, being the most abundant the diethoxylated species (NPEO_2_C); simultaneously, it is possible that the oxidation of the nonyl chain generates dicarboxylic compounds (CNPEO_1_C), without production of nonylphenol [12]. (III) Simultaneous shortening of the ethoxylated and alkylic chains to produce propyl and heptyl diethoxylated compounds [2] and (IV) oxidation of the polyethoxylated chain, without any shortening, to yield the corresponding carboxylic acids [27, 28]. Besides, it has been reported that the initial step in the NPEO_*x*_ degradation occurs on the ethoxylated moiety of the molecule, followed by further attack on the aromatic nonylphenol ring [29].

 The degradation of the aromatic ring occurs under aerobic conditions yielding several soluble metabolites which are finally degraded to CO_2_ [30]; the kinetic order of the reaction corresponds to a first-order process [31, 32]. In anaerobiosis, *Pseudomonas putida* degraded NPEO_*x*_, with different length chains (*x* = 6, 9 and 20), yielding as a final product NPEO_2_, no carboxylic acids were detected [8].

*Sphingomonas xenophaga* was able to cleave the aliphatic nonyl chain, bound to the phenol, depending on a specific branching pattern [5]; only the *para* isomer was degraded [23].

 Finally, degradation of NPEO_*x*_ is carried out by several bacterial genera: *Pseudomonas, Sphingomonas, Cupriavidus, Ralstonia, Achromobacter, Staphylococcus, Ochrobactrum, Castellania, Variovorax, Bacillus,* and *Psychrobacter* [25, 27, 28, 33–35].

Due to the negative effects of NPEO_*x*_ on animals and the natural environment, its use for manufacturing domestic detergents has been banned, and their industrial applications are seriously restricted in the European Community, United Kingdom, United States of America, New Zealand, and Japan [36]. However, in many countries, including Venezuela, China, and India [4], legislation to control the use of such surfactants is inexistent or is not realistic. Therefore, it seems necessary to carry out studies to search for indigenous bacterial strains able to degrade NPEO_*x*_ to be used as possible bioremediation agents on contaminated natural environments.

This paper describes the isolation of five NPEO_15_ degrading bacterial strains. Two of them, *Pseudomonas fluorescens* (strain Yas2) *and Klebsiella pneumoniae* (strain Yas1), showed high capacity for using NPEO_15_ as sole carbon source. The degradation process was followed by viscometry of the previously used culturing media and measurement of the chemical and biological oxygen demands. To our knowledge, this is the first report of the NPEO_*x*_ degrading activity by *Klebsiella* sp.

## 2. Materials and Methods

### 2.1. Biological Samples

The bacterial strains were isolated from soil samples collected in the gardens of the Centro de Investigaciones Microbiológicas Aplicadas (CIMA-UC), Campus Bárbula, Carabobo, Venezuela. To our knowledge these gardens are not contaminated with NPEO_15_.

### 2.2. Culturing Broth

The bacteria were grown in a minimal mineral medium (MM) containing the followings salts (p/v): 1% CuSO_4_, 0.1 mL; 0.5% FeSO_4_, 2 mL; 1% MgSO_4_, 0.2 mL; 1% ZnSO_4_, 0.5 mL; 0.7% NaCl, 0.05 mL; 0.1% NH_4_Cl, 1.0 mL; 0.8% NH_4_NO_3_, 0.125 mL and 1% CaCO_3_, 0.5 mL, volume was adjusted to 1.0 L with 0.1 M phosphate buffer at appropriated pH values, as it will be indicated further (MM medium). All salts were proanalysis quality and the ethoxylated nonylphenol (NPEO_15_, MW 880 g mol^−1^) was kindly donated by Palma Products, CA Valencia, Venezuela.

### 2.3. Culture of Bacteria (MM/NPEO_15_ Medium)

Soil samples (100 g) were suspended in MM medium (250 mL) in 500 mL flasks, at pH 7.0, in aerobiosis, and appropriate volumes of NPEO_15_ were added to reach final concentrations of 3% v/v and 30% v/v (0.0365 and 0.365 M, resp.). The systems were incubated at room temperature (22–25°C) under constant shaking during 45 days. Aliquots (3 mL), taken at different cultivation times, seeded in nutritive broth tubes and the bacterial growth was evaluated, after 24 h at 37°C, by single visual inspection of turbidity. After streaking of the total bacterial population on nutritive agar plates, after 24–48 h at 37°C, colonies were selected and assessed for growth in McConkey, Kliger's iron and oxidase media and finally the isolated colonies were stored in nutritive broth at 4°C until further use. The taxonomic identification was carried out by using the Analytical Profile Index (API) 20E and 20NE systems (bioMérieux sa, France).

### 2.4. Optimization of Bacterial Growth

The selected bacterial colonies were grown in 250 mL flasks containing MM/3% NPEO_15_ medium (50 mL), pH 7, in aerobiosis for 12 h at 27°C, and then aliquots were submitted to different experimental protocols.

- To assess the purity and viability of the colonies, aliquots (1 mL) were added to the same medium (150 mL) at 27°C and the bacterial growth was assessed, at intervals of 3 h, over a period of 9 h, by measuring the absorption at 660 nm on a Spectronic Genesys II spectrophotometer. Simultaneously, aliquots (1 mL) were used, to prepare solutions by successive dilution that allowed determining the number of colony forming units per milliliter (CFU mL^−1^).
- The MM/3% NPEO_15_ medium was adjusted to different pH values (7, 8, 8.5 and 9) and the bacterial growth was followed as indicated in protocol I.
- The MM medium was adjusted to 1, 3, and 5% NPEO_15_ concentrations (0.0121, 0.0365, and 0.0605 M, resp.). The bacterial growth was followed as indicated in protocol I.

### 2.5. Chemical Oxygen Demand (COD)

The assays were executed according to the open reflux method 5220B [37]. Bacterial strains were incubated for 24 h in MM/1% NPEO_15_ medium, 27°C, and pH 8, and bacterial growth was followed every 3 h, at 660 nm absorbance. Aliquots were withdrawn after 0, 6, and 24 h incubations, and the samples were filtered through Millipore membranes (0.45 *μ*m pore size) to obtain free bacterial filtrates (FBF) and submitted to analysis. A blank system (without bacterial inoculums) was also assayed. 

### 2.6. Biological Oxygen Demand (BOD5)

The assays were carried out according to the electrode membrane method 5210B [37]. After 24 h at 27°C, pH 8 in MM/1% NPEO_15_ medium, inoculums (1 Ml) were incubated in the same medium and conditions. Aliquots were withdrawn after 6, 24, and 48 h incubation times and dilutions prepared in water (300 mL) were incubated for 5 days at 20°C in a dark and dry chamber. The oxygen was determined by an OAKTON DO 100.A electrode system. The value at zero time was also assayed. 

### 2.7. Viscosity Studies

Volumes (5 mL) of bacterial cultures grown in MM/5% NPEO_15_ medium, pH 8, at 27°C for 12 h, were transferred to the same medium (300 mL) and aliquots were withdrawn at different times for one week at 25°C. Immediately after the removal, the samples were filtered through Millipore membranes (0.45 *μ*m pore size) to obtain the FBF and stored at −20°C until further use. The viscosity changes were determined in a Cannon 50W404 Ostwalt viscometer at 25°C and densities in a 10 mL pycnometer. A calibration curve relating flow times (seconds) in the viscometer to NPEO_15_ concentrations (mM) was obtained in order to determine the remaining NPEO_15_ concentrations. Values, at 25°C, for water density (0.99704 g mL^−1^) and viscosity (0.8904 g cm^−1^ s^−1^, centipoises) were taken from Weast [38]. Relative viscosity values were calculated by the expression *η*_*s*_ = *η*_*o*_ *t*_*s*_ *ρ*_*s*_/*t*_*o*_ *ρ*_*o*_, where zero subscript refers to water and *s* to NPEO_15_ aqueous solutions [39, 40].

## 3. Results and Discussion

### 3.1. Bacterial Colony Isolation and Taxonomic Identification

From the soil samples five indigenous bacterial colonies were isolated, of which strain H grew on MM/3% NPEO_15_ medium and the other four (strains F, K, N, and O) on MM/30% NPEO_15_.  Table 1 shows the morphological and biochemical characteristics of the isolated strains. 

**Figure 1:**
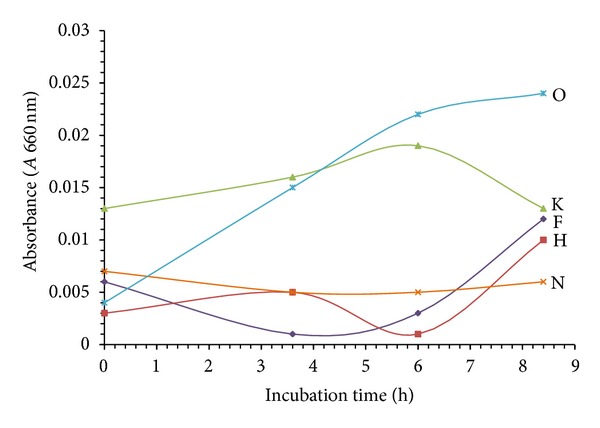
Growth of bacterial strains isolated from soil.

The bacterial growth on MM/3% NPEO_15_ medium, pH 7, 27°C is shown in Figure 1. Strains K and O showed the highest growth rates; both grew without lag phase and the logarithmic phase was observed until 6 h incubation time. Because of this behavior both strains were chosen to perform subsequent experiments. According to the API identification system strains K and O were identified as *Pseudomonas fluorescens* and *Klebsiella pneumonia *and were named Yas2 and Yas1, respectively.

### 3.2. Bacterial Growth Conditions

**Figure 2:**
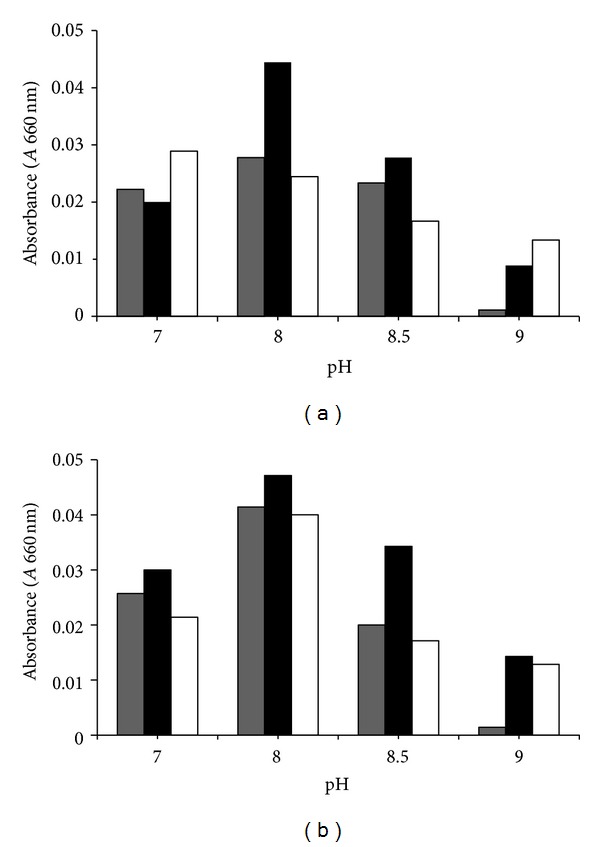
Bacterial growth on 3% NPEO_15_ at different pH values. Gray, black, and white bars indicate bacterial growth at 3, 6, and 9 h, respectively. (a) *Klebsiella pneumoniae* strain Yas1; (b) *Pseudomonas fluorescens* strain Yas2.

The behavior of the bacterial strains at different pH values is described in Figure 2. Maximal growth rates were achieved at pH 8 (0.04 and 0.033 absorbance units), whereas the minimal growth was observed at pH 9 (0.008 and 0.01 au) after 6 h culturing time. These data correlated well with the CFU mL^−1^ numbers at 6 h culture: 10^6^–10^7^ and 10^3^–10^4^ CFU mL^−1^ at pH 8 and 9, respectively (data not shown).

**Figure 3:**
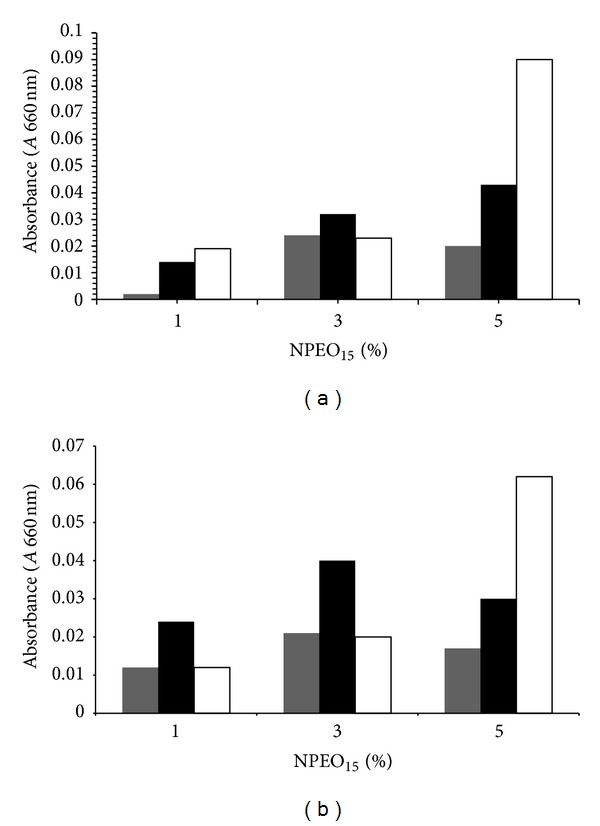
Bacterial growth at different NPEO_15_ concentrations. Grey, black, and white bars indicate bacterial growth at 3, 6, and 9 h, respectively. (a) *Klebsiella pneumoniae* strain Yas1. (b) *Pseudomonas fluorescens* strain Yas2.

In Figure 3 the bacterial growth patterns with respect to the NPEO_15_ concentration in the media are shown. Maximal (0.09 and 0.062 au) and minimal (0.019 and 0.012 au) growth values were achieved at 5% and 1% NPEO_15_, respectively, at 9 h incubation times. 

**Figure 4:**
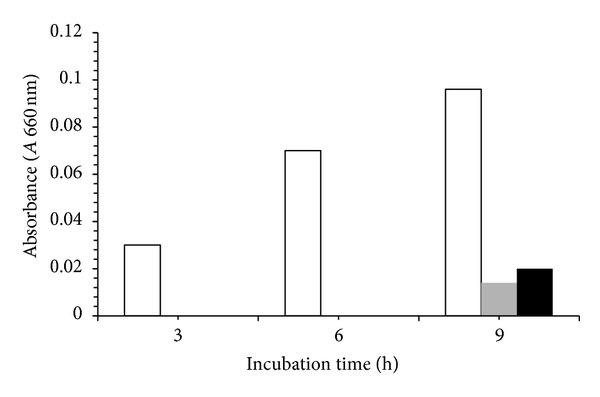
Growth of a mixture culture of Yas1/Yas2 on 1% NPEO_15_. Grey and Black colors correspond to the individual cultures of Yas1 and Yas2 respectively.

Usually, in the culture media of 1% and 3% the maximal growth was observed at 6 h incubation, and then it reached the stationary growth phase at 9 h. The logarithmic growth phase persisted until 9 h for the 5% NPEO_15_ system, and then the stationary phase disappeared. Finally, a mixture culture of Yas1/Yas2 in 1% NPEO_15_ medium, pH 8, showed a synergistic effect on the bacterial growth, reaching maximal values (0.095 au) at 9 h incubations and 27°C, which represents a stimulatory average factor close to 5.7 with respect to the individual bacterial cultures at 1% NPEO_15_ medium (0.014 and 0.02 au); additionally, the lag phase was absent (see Figure 4). This stimulatory effect is probably due to NPEO_*x*_ cometabolism by the Yas1/Yas2 system, as it has been described for other bacterial consortiums [41].

### 3.3. Degradation of NPEO_15_ by Bacteria

**Figure 5:**
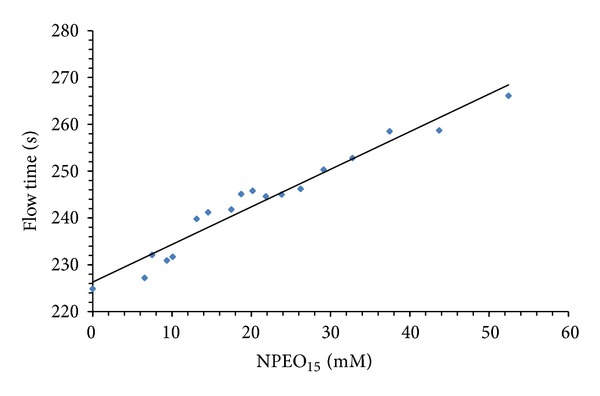
Calibration curve relating flow time to NPEO_15_ concentration. Each point represents the average of three determinations.

The NPEO_15_ biodegradation was determined in FBF by following the flow time in a viscometer and by the chemical oxygen demand, also the biological oxygen demand was evaluated.  Figure 5 shows the calibration curve that relates the molarity of NPEO_15_ solutions and their flow times in a viscometer. The data fit the equation *Y* = 226.9 + 0.9418*X*  (*R* ^2^ = 0.94) calculated by linear regression. 

Tables 2 and 3 show the viscosity and density changes, flow times (s), molarity (M) of remaining NPEO_15_, and density (g L^−1^) and viscosity (g cm^−1^ s^−1^) of the FBF corresponding to different incubation times for both bacterial strains. A decrease in the FBF viscosity and density correlated well to the observed decrease of the determined flow times. From an initial value of 1.150 g cm^−1^ s^−1^ the viscosity was reduced to 0.8959 and 0.8490 for Yas1 and Yas2 systems, respectively. These facts indicated the cleavage of the NPEO_15_ to lower molecular weight species. On basis of these data Yas1 and Yas2 degraded 0.0392 (0.0605–0.0213) and 0.0383 NPEO_15_ moles L^−1^ (0.0605–0.0216) in 54.5 and 134 h, respectively. The extension of the degradation process has been reported as temperature dependent [6, 42]. In this study degradation was close to 65% at 27°C; similar values have been reported by other authors [6, 40, 41, 43]. 

It was also observed that foam appearing at the beginning of the incubations decreased at late incubation times. At 71 h (Yas1) and 163 h (Yas2) incubations, foam had totally disappeared and simultaneously, viscosity increases were evident in both cultures. It is well known that several bacterial genera are able to produce viscous polymers (mucopolysaccharides, dextrans, proteins, poly-*β*-hydroxybutyrate, polyphosphates, and xanthans) as strategies to retain nutrients and water, as energy reserves and for defense purposes [44–48]. Another aspect for consideration is the observed viscosity changes with the incubation times; Yas1 decreased the viscosity from 1.15 g cm^−1^ s^−1^ to 0.8959 in 54.5 h, a relative short time; at 8.5 h of incubation the viscosity fell, but it suddenly rose (22.5 h) to fall again at 32.5 h incubation. This apparent data dispersion could be due to the well-known mucogenic properties of the *Klebsiella* genus [44, 46, 47], which was also observed in this study (see Table 1). On the other hand, Yas2 changed the viscosity from 1.15 to 0.849 g cm^−1^ s^−1^, in 134 h, a relative longer time, without abrupt changes. Thus, Yas1 produced viscous materials at early and late incubation times, whereas Yas2 did it only at late times. Synthesis of dextrans and alginates by *Klebsiella* and *Pseudomonas*, respectively, has been reported [49–51]; therefore, viscometry studies to evaluate degradation of viscous substances seem to be inappropriate when the bacteria are capable of synthesizing viscous polymers during the whole incubation time, such as *Klebsiella *did. However, if the bacteria yield viscous molecules at relatively late times during the incubation, then the viscosity changes could be a useful, cheap, and rapid method to detect biodegradation of viscous xenobiotic polymers, as in the *Pseudomonas* system. Regardless of whether the bacteria degrade NPEO_15_ or the bacterial polymers, differences in degradation times displayed by the bacterial strains would imply that Yas1 cleaved chemical bonds near the aromatic ring in the NPEO_15_ molecule, producing low molecular weight species and causing a rapid change in the viscosity, whereas Yas2 seems to exert its action progressively on bonds near to the hydroxylated end of the surfactant ethoxylated chain, thus the decrease of the molecular mass was not as abrupt and the viscosity decrease should be slow. An alternative possibility is to assume Yas1 is a better enzyme producer. The reported data in Tables 2 and 3 allowed obtaining kinetic information about the NPEO_15_ biodegradation. Taking the initial (*C*_*o*_) and remaining (*C*_*x*_) NPEO_15_ concentrations at different incubation times it was possible to determine the kinetic order for the NPEO_15_ biodegradation according to first- and second-order kinetic equations for a chemical reaction [39], also the kinetic rate constant *k* and the half life time *τ* for the NPEO biotransformation were calculated. 

**Figure 6:**
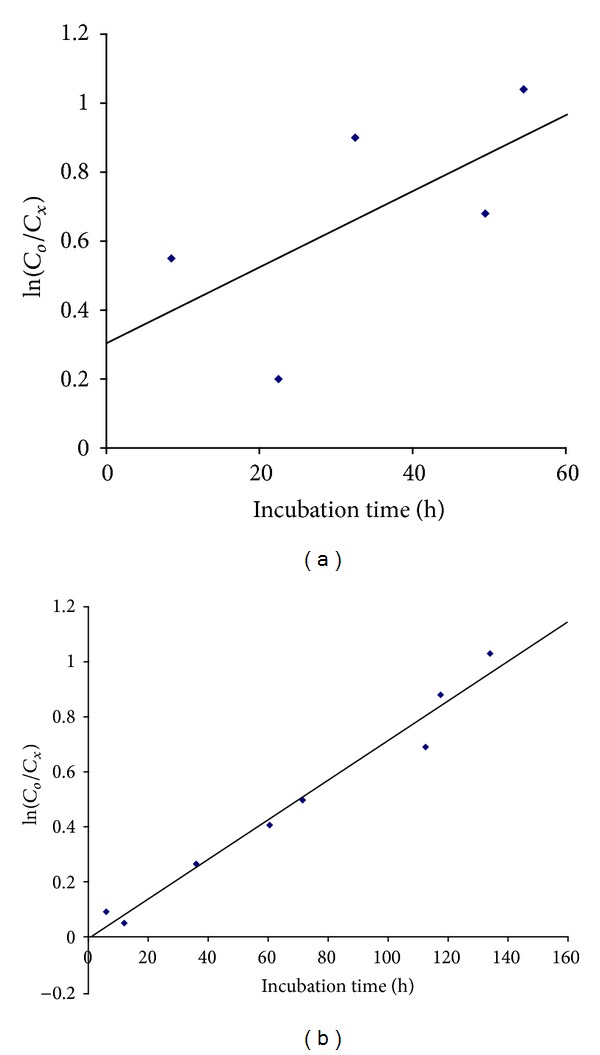
Kinetic order for the NPEO_15_ biodegradation reaction. (a) *Klebsiella pneumoniae* strain Yas1 (*Y* = 0.309 + 0.0109*X*;  *R* ^2^ = 0.4028); (b) *Pseudomonas fluorescens* strain Yas2 (*Y* = 0.0072*X* − 0.0053;  *R* ^2^ = 0.9728).

Figure 6(b) shows the ln⁡(*C*_*o*_/*C*_*x*_) versus *t* plot corresponding to the Yas2 data (Table 3). The obtained straight line (*Y* = 0.0072*X*–0.0053,  *R* ^2^ = 0.9728) indicated the NPEO_15_ biodegradation obeyed first-order reaction kinetics; data from other laboratories indicated that NPEO_1_ and NPEO_2_ also obeyed the same order kinetics [32]. A similar analysis for the Yas1 system (Figure 6(a)) did not allow assigning any reaction order because of the viscous material synthesized at early and late time incubations by the Yas1 strain. The estimated values of the rate constant *k* and time *τ* for Yas2 were 0.0072 h^−1^ and 96.3 h, respectively. For these calculations the corresponding data at 163 h were not considered because the observed viscosity increments were probably due to the synthesis of viscous bacterial polymers and not due to NPEO_15_ present in the media.

Although the maximal bacterial growth was obtained at 5% NPEO_15_, the following experiments were executed at 1% NPEO_15_ because the principal aim of this study was to propose a satisfactory solution to the real environmental NPEO contamination which according to several reports should be less than 1 mg L^−1^ [23, 52–55].

The chemical and biological oxygen demands, determined in broths previously used by microorganisms, are indirect measurements of the carbonaceous substrate degradation by a microbial population. Tables 4 and 5 shows the chemical oxygen demand (COD) and the biological oxygen demand (BOD) of both bacterial cultures in MM/1% NPEO_15_ medium, pH 8 at different incubation times. 

After 24 h incubation, the COD values decreased from an initial value of 20,230 mgO_2_ L^−1^ (zero time) to 666 and 3,066 mgO_2_ L^−1^, which represent 96 and 85% NPEO_15_ degradation for Yas1 and Yas2 strains, respectively (see Table 4). These low COD values, representing 4 and 15% of the initial values, indicate that both bacterial strains can probably degrade NPEO_15_ and its low ethoxylated derivatives, including nonylphenol, as it has been reported for other bacterial strains [6, 26, 43, 56]. The BOD_5_ data (Table 5) indicated that both bacterial strains consumed 99% (60.11/8,675 and 67.8/14,000) and 99.9% (5.26/8,675 and 5.11/14,000) of the available oxygen in 24 and 48 h, respectively, which implies that the NPEO_15_ derivatives did not exert appreciable toxic effects on the bacterial strains and are biodegradable. 

NPEO_*x*_ and its derivative degrading bacterial strains have been isolated from several natural environments and wastewater treatment plants [1, 5, 21, 30, 57–59]. In this paper the reported data indicated that indigenous bacterial strains, isolated from soil, are able to use NPEO_15_ as the sole carbon source. Although the viscosity descent is indicative of bond cleavages in the NPEO_15_ molecule, it does not imply the use of the degradation products for sustaining the bacterial viability. However, the changes in the chemical and biological oxygen demands and the increments (viability) in the CFU mL^−1^ number in NPEO_15_ complemented media indicate that Yas1 and Yas2 must use the NPEO_15_ degradation products to satisfy their metabolic requirements and support cellular division. *P. fluorescens* (Yas1) and* K. pneumoniae* (Yas2) thus appear to be useful biotechnological tools to bioremediate NPEO contaminated waters and soils. 

## 4. Conclusions

Five bacterial strains, isolated from soil, grew on a minimal mineral medium supplemented with NPEO_15_ (0.0365 M and 0.365 M) as the sole carbon source, being *Pseudomonas fluorescens* and *Klebsiella pneumoniae* the two most efficient NPEO_15_ degrading strains. The extent of NPEO_15_ degradation after 24–48 h incubations, evaluated by COD and BOD_5_ assays, was, 85–95% and 99.9% respectively. The kinetic rate constant (*k*) and the half life time (*τ*) for the NPEO_15_ biotransformation by *P. fluorescence* were estimated to be 0.0072 h^−1^ and 96.3 h, respectively, and the process followed first-order kinetics.

## Figures and Tables

**Table 1:** Morphological and biochemical characteristics of bacterial strains isolated from soil.

Strain	Morphology	Gram	MacConkey	Kliger iron's	Oxidase
F	Red colonies Large and thin bacilli	Negative	Lactose+	Glucose+	−
H	Light yellow colonies Large and thin bacilli	Negative	Lactose−	Glucose−	+
K Yas1	Dark yellow, mucous colonies Small bacilli	Negative	Lactose+	Glucose+	−
N	Brown colonies Small and thin bacilli	Negative	Lactose−	Glucose−	+
O Yas2	Ligth yellow colonies Small bacilli	Negative	Lactose−	Glucose−	+

Signs indicate positive/negative fermentation or enzymatic activity.

**Table 2:** Physical chemical parameters of the 0.0605 M NPEO_15_ degradation by *Klebsiella pneumoniae* strain Yas1.

Incubation time (h)	Flow time (s)*	Remaining NPEO (moles L^−1^)	Density (g L^−1^)**	Viscosity (g cm^−1^ s^−1^)
0	283.9	0.0605	1.0200	1.1500
8.5	259.7 ± 1.689^†^	0.0348	0.9287 ± 0.0395^†^	0.9583
22.5	273.6 ± 1.376	0.0495	0.9964 ± 0.044	1.0831
32.5	250.0 ± 2.185	0.0245	0.9146 ± 0.0049	0.9084
49.5	256.0 ± 1.260	0.0308	0.9140 ± 0.0071	0.9296
54.5	247.0 ± 2.753	0.0213	0.9129 ± 0.0058	0.8959
71.0	285.5 ± 1.941	0.0622	1.0137 ± 0.039	1.1479

*Flow time of the FBF in the viscometer at 25°C, average of nine measurements. **Average of six measurements. ^†^Standard deviation.

**Table 3:** Physical chemical parameters of the 0.0605 M NPEO_15_ degradation by *Pseudomonas fluorescens* strain Yas2.

Incubation time (h)	Flow time (s)*	Remaining NPEO (moles L^−1^)	Density (g L^−1^)**	Viscosity (g cm^−1^ s^−1^)
0	283.9	0.0605	1.0200	1.1500
6.0	278.9 ± 1.969^†^	0.0552	1.0064 ± 0.001^†^	1.1152
12.0	280.9 ± 2.320	0.0573	1.0067 ± 0.002	1.1235
36.0	270.6 ± 1.056	0.0464	1.0141 ± 0.0047	1.0903
60.5	264.9 ± 1.357	0.0403	0.9805 ± 0.0132	1.0320
71.5	261.6 ± 1.069	0.0368	0.9098 ± 0.0029	0.9456
112.5	255.4 ± 0.853	0.0302	0.9190 ± 0.0053	0.9325
117.5	251.3 ± 0.506	0.0259	0.8714 ± 0.0365	0.8700
134.0	247.3 ± 2.573	0.0216	0.8641 ± 0.0216	0.8490
163.0	285.9 ± 1.354	0.0626	0.9855 ± 0.0488	1.1194

*Flow time of the FBF in the viscometer at 25°C, average of six measurements. **Average of four measurements. ^†^Standard deviation.

**Table 4:** Chemical oxygen demand.

Incubation time (h)	COD values (mg O_2_ L^−1^)	
	Strain Yas1	Strain Yas2
0	20,230 ± 4,303^†^	20,230 ± 4,303^†^
6	2,000 ± 1,058	3,333 ± 1,890
24	666.4 ± 462	3.066 ± 1.285

^†^Standard deviation.

**Table 5:** Biological oxygen demand.

Incubation time (h)	BOD values (mg O_2_ L^−1^)	
	Strain Yas1	Strain Yas2
0	8,675	14,000 ± 5,515^†^
6	2,245 ± 1,874^†^	1,992 ± 1,295
24	60.11 ± 51.3	67.8 ± 46.9
48	5.26 ± 4.12	5.11 ± 3.11

^†^Standard deviation.

## References

[1] Manzano-Quiones MA, Vargas-Machuca JAP, Sales-Mrquez D, Quiroga-Alonso JM (1998). Cinética de biodegradacin de un nonilfenol polietoxilado en agua de ro. *Ingeniería del Agua*.

[2] Jiménez-González A, Siles-Alvarado S, Monroy O (2003). Biodegradation of octylphenol polyethoxylates by denitrification. *Water Science and Technology*.

[3] Vzquez-Duhalt R, Mrquez-Rocha F, Ponce E, Licea AF, Liana MT (2005). Nonylphenol an integrated vision of a pollulant. *Applied Ecology and Environmental Research*.

[4] Mao Z, Zheng X-F, Zhang Y-Q, Tao X-X, Li Y, Wang W (2012). Occurrence and biodegradation of nonylphenol in the environment. *International Journal of Molecular Sciences*.

[5] Gabriel FLP, Giger W, Guenther K, Kohler H-PE (2005). Differential degradation of nonylphenol isomers by *Sphingomonas xenophaga* Bayram. *Applied and Environmental Microbiology*.

[6] Ying G-G (2006). Fate, behavior and effects of surfactants and their degradation products in the environment. *Environment International*.

[7] Tanghe T, Devriese G, Verstraete W (1998). Nonylphenol degradation in lab scale activated sludge units is temperature dependent. *Water Research*.

[8] John DM, White GF (1998). Mechanism for biotransformation of nonylphenol polyethoxylates to xenoestrogens in *Pseudomonas putida*. *Journal of Bacteriology*.

[9] Correa-Reyes G, Viana MT, Marquez-Rocha FJ, Licea AF, Ponce E, Vazquez-Duhalt R (2007). Nonylphenol algal bioaccumulation and its effect through the trophic chain. *Chemosphere*.

[10] Soares A, Guieysse B, Jefferson B, Cartmell E, Lester JN (2008). Nonylphenol in the environment: a critical review on occurrence, fate, toxicity and treatment in wastewaters. *Environment International*.

[11] Giger W, Brunner PH, Schaffner C (1984). 4-Nonylphenol in sewage sludge: accumulation of toxic metabolites from nonionic surfactants. *Science*.

[12] Voogt P, Kwast O, Hendriks R, Jonkers N (2000). Alkylphenol ethoxylates and their degradation products in abiotic and biological samples from the environment. *Analusis*.

[13] Toyooka T, Kubota T, Ibuki Y (2012). Nonylphenol polyethoxylates induce phosphorylation of histone H2AX. *Mutation Research*.

[14] Marcomini A, Pavoni B, Sfriso A, Orio AA (1990). Persistent metabolites of alkylphenol polyethoxylates in the marine environment. *Marine Chemistry*.

[15] Ahel M, McEvoy J, Giger BW (1993). Bioaccumulation of the lipophilic metabolites of nonionic surfactants in freshwater organisms. *Environmental Pollution*.

[16] Adam FIM, El-Ashry ZM (2010). Evaluation of genotoxicity of 4-n-nonylphenol using *Vicia fabal* L. *Journal of Biological Sciences*.

[17] Frassinetti S, Barberio C, Caltavuturo L, Fava F, Di Gioia D (2011). Genotoxicity of 4-nonylphenol and nonylphenol ethoxylate mixtures by the use of *Saccharomyces cerevisiae* D7 mutation assay and use of this text to evaluate the efficiency of biodegradation treatments. *Ecotoxicology and Environmental Safety*.

[18] Tam NFY, Wang P, Gao QT, Wong YS Toxicity of waterborne persistent organic pollulants on green microalgae.

[19] Sayed AEDH, Hakeem SSA, Mahmoud UM, Mekkawy IA (2012). 4 nonylphenol induced morphological and histopathological malformation in Bufo regularis tadpoles. *Global Advanced Research Journal of Environmental Science and Toxicology*.

[20] Karahan Ö, Olmez-Hanci T, Arslan-Alaton I, Orhon D (2010). Modelling biodegradation of nonylphenol ethoxylate in acclimated and non-acclimated microbial cultures. *Bioresource Technology*.

[21] Ferguson PL, Brownawell BJ (2003). Degradation of nonylphenolethoxylates in estuarium sediment under aerobic and anaerobic conditions. *Environmental Toxicology and Chemistry*.

[22] Lu J, He Y, Wu J, Jin Q (2009). Aerobic and anaerobic biodegradation of nonylphenol ethoxylates in estuary sediment of Yangtze River, China. *Environmental Geology*.

[23] Tanghe T, Dhooge W, Verstraete W (1999). Isolation of a bacterial strain able to degrade branched nonylphenol. *Applied and Environmental Microbiology*.

[24] Andrade-Ribeiro A ALF, Pacheco-Ferreira A, Nbrega da Cunha CL, Mendes-Kling AS (2006). Disruptores endocrinos: potencial problema para la salud pblica y medio ambiente. *Revista Biomédica*.

[25] Maki H, Masuda N, Fujiwara Y, Ike M, Fujita M (1994). Degradation of alkylphenol ethoxylates by *Pseudomonas sp.* strain TR01. *Applied and Environmental Microbiology*.

[26] Liu X, Tani A, Kimbara K, Kawai F (2006). Metabolic pathway of xenoestrogenic short ethoxy chain-nonylphenol to nonylphenol by aerobic bacteria, *Ensifer* sp. strain AS08 and *Pseudomonas* sp. strain AS90. *Applied Microbiology and Biotechnology*.

[27] Gu X, Zhang Y, Zhang J (2010). Isolation of phylogenetically diverse nonylphenol ethoxylate-degrading bacteria and characterization of their corresponding biotransformation pathways. *Chemosphere*.

[28] Gu X, Zhang Y, Zhang J, Yang M, Takaki H, Kamala Y (2008). Degradation behaviors of nonylphenolethoxylates by isolated bacteria using improved isolation method. *Journal of Environmental Sciences*.

[29] McAdam EJ, Bagnall JP, Soares A (2011). Fate of alkylphenolic compounds during activated sludge treatment: impact of loading and organic composition. *Environmental Science and Technology*.

[30] Wang P, Nong X-H, Ge J-H (2011). Aerobic biodegradation of nonylphenol ethoxylates in shaking-flask test. *Electronic Journal of Biotechnology*.

[31] Naylor CG, Williams JB, Varineau PT Biodegradation of the ^14^C ring labeled nonylphenolethoxylates in activated sludge and in river water.

[32] Qiao Y-S, Zhang J, Yang M, Zhang Y, Xu D-Y (2008). Degradation of nonylphenol and short chain nonylphenol polyethoxylates in soil. *Huang Jing Ke Xue*.

[33] Di Gioia D, Michelles A, Pierini M, Bogialli S, Fava F, Barberio C (2008). Selection and characterization of aerobic bacteria capable of degrading commercial mixtures of low-ethoxylated nonylphenols. *Journal of Applied Microbiology*.

[34] Tuan NN, Hsieh H-C, Lin Y-W, Huang S-L (2011). Analysis of bacterial degradation pathways for long-chain alkylphenols involving phenol hydroxylase, alkylphenol monooxygenase and catechol dioxygenase genes. *Bioresource Technology*.

[35] Yang G, Zhang Y, Bai Y (2011). Purification and characterization of a nonylphenol (NP)-degrading enzyme from *Bacillus cereus*. Frankland. *Chinese Journal of Chemical Engineering*.

[36] Tremblay LA, Sterwart M, Peake BM, Gadd JB (2011). Review of the risks of emerging organic contaminants and potential impacts to Hawke's Bay.

[37] (1998). *Standard Methods for the Examination of Water and Wastewater*.

[38] Weast RC (1987). *Handbook of Chemistry and Physics*.

[39] Castellan GW *Fisicoqumica, Segunda Edicin*.

[40] van Holde KE (1971). *Physical Biochemistry*.

[41] Song W, Lim KS, Yu DU (2011). Isolation of a nonylphenol-degrading microbial consortium. *Korean Journal of Fisheries and Aquatic Sciences*.

[42] Manzano MA, Perales JA, Sales D, Quiroga JM (1999). The effect of temperature on the biodegradation of a nonylphenol polyethoxylate in river water. *Water Research*.

[43] Xie Y, Zhu T, Liu X, Liu H, Han J (2013). Degradation of nonylphenol polyethoxylates and its microflora structure in an anoxin-oxic activated sludge process. *Adv Mat Res*.

[44] Holt JG, Krieg NR, Sneath PHA, Staley JT, Williams ST (1994). *Bergey's Manual of Determinative Bacteriology*.

[45] Madigan MT, Martinko JM, Parker, Brock J (2004). *Biologa De Los Microorganismos*.

[46] McFaddin J (2004). *Pruebas Bioqumicas Para La Identificacin De Bacterias De Importancia Clnica*.

[47] Forbes BA, Sahm DF, Wissfeld AS (2004). *Bailey and Scott Diagnstico Microbiolgico*.

[48] Vu B, Chen M, Crawford RJ, Ivanova EP (2009). Bacterial extracellular polysaccharides involved in biofilm formation. *Molecules*.

[49] Wingender J, Neu TR, Flemming HC, Wingender J, Neu TR, Fleming HC (1999). What are bacterial extracellular polymeric substances. *Microbial Extracellular Polymeric Substances: Characterization, Structure and Function*.

[50] Bu'lock J, Kristiansen BJ (1991). *Biotecnologa Bsica*.

[51] Feng L, Li X, Du G, Chen J (2009). Characterization and fouling properties of exopolysaccharide produced by Klebsiella oxytoca. *Bioresource Technology*.

[52] Ferguson PL, Iden CR, Brownawell BJ (2001). Distribution and fate of neutral alkylphenol ethoxylate metabolites in a sewage-impacted urban estuary. *Environmental Science and Technology*.

[53] Komori K, Okayasu Y, Yasojima M, Suzuki Y, Tanaka H (2006). Occurrence of nonylphenol, nonylphenol ethoxylate surfactants and nonylphenol carboxylic acids in wastewater in Japan. *Water Science and Technology*.

[54] Spehar RL, Brooke LT, Markee TP, Kahl MD (2010). Comparative toxicity and bioconcentration of nonylphenol in freshwater organisms. *Environmental Toxicology and Chemistry*.

[55] He F, Niu L, Aya O, Wang S, Wang L (2013). Ocurrence and fate of nonylphenol ethoxylates and their derivatives in Nansi Lake environments, China. * Water Environment Research*.

[56] Salvadori L, Di Gioia D, Fava F, Barberio C (2006). Degradation of low-ethoxylated nonylphenols by a *Stenotrophomonas* strain and development of new phylogenetic probes for *Stenotrophomonas* spp. detection. *Current Microbiology*.

[57] Peng X, Wang Z, Mai B (2007). Temporal trends of nonylphenol and bisphenol A contamination in the Pearl River Estuary and the adjacent South China Sea recorded by dated sedimentary cores. *Science of the Total Environment*.

[58] Li F, Tsumori J, Suzuki Y, Tanaka H (2008). Vertical distribution of nonylphenol ethoxylates and their derivatives in sediments of a freshwater reservoir. *Water, Air, and Soil Pollution*.

[59] Yu DU (2011). Isolation and characterization of nonylplhenol degrading bacteria. *Fisheries and Aquatic Sciences*.

